# Assessing the benefits of anaortic off-pump coronary artery bypass grafting

**DOI:** 10.3389/fcvm.2024.1393921

**Published:** 2024-05-07

**Authors:** Ryohei Ushioda, Aina Hirofuji, Dit Yoongtong, Boonsap Sakboon, Jaroen Cheewinmethasiri, Thanin Lokeskrawee, Jayanton Patumanond, Suppachai Lawanaskol, Hiroyuki Kamiya, Nuttapon Arayawudhikul

**Affiliations:** ^1^ Cardiovascular and Thoracic Surgery Unit, Department of Surgery, Lampang Hospital, Lampang, Thailand; ^2^ Department of Cardiac Surgery, Asahikawa Medical University, Asahikawa, Japan; ^3^ Department of Emergency Medicine, Lampang Hospital, Lampang, Thailand; ^4^ Center for Clinical Epidemiology and Clinical Statistics, Faculty of Medicine, Chiang Mai University, Chiang Mai, Thailand; ^5^ Chaiprakarn Hospital, Chiang Mai, Thailand

**Keywords:** anaortic, aorta no-touch technique, off-pump coronary artery bypass grafting, stroke, propensity score matching

## Abstract

**Introduction:**

The procedure called the “aorta no-touch” (NT) or anaortic technique in off-pump coronary artery bypass grafting (OPCAB) is designed to reduce the perioperative risk of stroke. We have observed an increased frequency of anaortic OPCAB procedures at our institution. The main purpose of the present study is to investigate the effectiveness of anaortic OPCAB in reducing the perioperative risk of stroke.

**Methods:**

From April 2011 to July 2023, a total of 2,236 patients underwent isolated OPCAB at our single center. The patients were divided into the anaortic group (NT, *n* = 762) and the aortic group (A, *n* = 1,474). The NT group was propensity score-matched (PSM) with the A group at a 1:1 ratio (NT *n* = 640; A *n* = 640), and matching was performed based on 26 covariates with preoperative clinical characteristics.

**Results:**

In both the unmatched and matched cohorts of the NT and A groups, there were no significant differences observed in new stroke rates (NT vs. A; unmatched, 1.0% vs. 1.2%, *p* = 0.624; matched, 0.9% vs. 1.3%, *p* = 0.789). The univariable logistic analysis did not identify the anaortic technique as an independent factor negatively associated with new stroke events (OR = 0.81, 95% CI = 0.35–1.86, *p* = 0.624).

**Conclusion:**

The present study did not find the anaortic technique to reduce the perioperative risk of stroke in OPCAB. Hence, further large studies are needed to identify patient cohorts in which anaortic OPCAB is significantly beneficial.

## 1 Introduction

Numerous studies have investigated the outcomes, advantages, and challenges associated with off-pump coronary artery bypass grafting (OPCAB) performed with the aorta no-touch (anaortic) technique (1, 2). This strategic approach notably reduces the perioperative risk of stroke (3), yet it demands advanced techniques such as harvesting skeletonized arterial grafts to ensure sufficient length, the creation of composite grafts, and precise graft alignment during sequential bypass. Despite its patient-centric advantages, the adoption of this technically demanding procedure remains limited, with only a handful of cardiac surgeons venturing into this style of operation, likely owing to its significant learning curve (4). Our department has routinely performed OPCAB for patients with coronary artery diseases since 2011 and recently shifted to performing anaortic OPCAB with more frequency in cases that appear theoretically and anatomically reasonable. This study investigated whether anaortic OPCAB was more effective for stroke reduction.

## 2 Patients and methods

From April 2011 to July 2023, a total of 2,236 patients underwent isolated OPCAB in our center. These patients were divided into two groups based on the presence or absence of aortic manipulation, namely, the anaortic group [“aorta no-touch” (NT), *n* = 762] and the aortic group (A, *n* = 1,474). Univariable or multivariable logistic regression analyses were used to identify independent prognostic factors of a new stroke. In addition, the NT group was propensity score-matched (PSM) with the A group at a 1:1 ratio (NT, *n* = 640; A, *n* = 640), and matching was performed based on 26 covariates of preoperative clinical characteristics. After matching, patient characteristics, preoperative evaluation details, operative procedures, and postoperative outcomes were compared between both groups. The institutional review board of Lampang Hospital approved this retrospective study and waived the need for written patient consent. A new stroke was defined as the development of a new focal neurologic deficit confirmed by clinical findings and a computed tomography (CT) scan within the duration of the patient’s hospital stay. Strokes occurring after discharge were defined as long-term stroke events. In postoperative management, dual antiplatelet therapy was prescribed as discharge medication.

### 2.1 Surgical procedure and OPCAB strategy at our institution

All patients in the present study underwent OPCAB. Our institution has a total of five surgeons, each of whom performed anaortic OPCAB. The operative procedures were as follows. The approach was through median sternotomy or left mini-thoracotomy, and the target vessel for each anastomosis was appropriately exposed using a tissue stabilizer (Octopus tissue stabilizer, Medtronic, Minneapolis, MN, USA) with or without a deep pericardial stitch or a heart positioner such as the Starfish heart positioner (Medtronic). Graft selection and bypass design were determined by the primary surgeon, and complete revascularizations of the major coronary artery branches were achieved in every case. Prior to arteriotomy, stitches were placed in both the proximal and distal regions of the target vessels using elastic silicone tubing or monofilament suture material. 8-0 polypropylene sutures were used for anastomosis with the internal thoracic artery, while 7-0 polypropylene sutures were used for anastomosis with the gastroepiploic or radial artery. Our institution prioritizes the anaortic approach for OPCAB when it appears theoretically and anatomically suitable. Anaoritc OPCAB was performed in 34.1% (*n* = 762) of all OPCAB cases, mainly in patients with all arterial revascularisation, left mini-thoracotomy including minimally invasive direct coronary artery bypass grafting (MIDCAB), and preoperative CT showing severe aortic calcification. In OPCAB with aortic manipulation (*n* = 1,414), aortic side clamping was mainly utilized for proximal anastomosis onto the ascending aorta, and an anastomosis assist device (Guidant Heartstring, Guidant Corporation, Santa Clara, CA, USA, *n* = 5; Enclose II, Novare Surgical Systems, Cupertino, CA, USA, *n* = 55) was used sporadically. In patients who present with poor preoperative conditions or require multiple distal anastomoses, we consider performing the aorta-coronary bypass technique by saphenous vein. However, in patients with a history of cerebrovascular events, we actively opt for the anaortic approach.

Preoperative CT scans were only performed in patients aged over 75 years and those suspected of aortic risk based on their x-rays. In postoperative management, dual antiplatelet therapy was prescribed as discharge medication. The use of anticoagulants was not standard practice in our study population. However, in patients who developed atrial fibrillation (AF) a few days postoperatively, warfarin was prescribed, and prothrombin time international normalized ratio (PT INR) levels were monitored and maintained within the range of 2–3.

### 2.2 Follow-up

The patients were followed up every 6 months at our outpatient clinic. Information on all causes of death and cardiac complications during the follow-up period was obtained from Lampang Hospital's database. We achieved a 100% follow-up rate by contacting both the patients and their families for any missing data.

### 2.3 Statistical analysis

Group assignments were not randomized because the operative approach was a matter of subjective choice. Therefore, we calculated standardized mean differences before and after PSM to assess the balance of variables between the groups. The propensity score (PS) was obtained from a logistic regression model, including 23 covariables presented in Table 1, excluding the European System for Cardiac Operative Risk Evaluation (Euro SCORE) Ⅱ, Society of Thoracic Surgeons (STS) score, and emergent case (Table 2). Patients were matched at a 1:1 ratio using the nearest neighbor matching method without replacement and a caliper width of 0.2 of the standard deviation of the logit of the estimated PS. The continuous variables exhibiting a normal distribution were tested using the *t*-test, and the continuous variables exhibiting a non-normal distribution were tested using the Mann–Whitney *U*-test. For the categorical variables, McNemar's test was conducted in the matched cohort, while Fisher's exact test and chi-square test were performed in the unmatched cohort. Statistical significance was set at *p* < 0.05. The Kaplan–Meier method was used to demonstrate survival rate and freedom from major adverse cardiac or cerebrovascular events (MACCE). The STATA software/MP version 17.0 (Stata Corporation, College Station, TX, USA) was used for the statistical analyses.

**Table 1:** Patient characteristics and preoperative data.

	Entire cohort				Matched cohort			
	NT group (*n* = 762)	A group (*n* = 1,474)	*p*-value	SMD	NT group (*n* = 640)	A group (*n* = 640)	*p*-value	SMD
Age, mean ± SD years	64.6 ± 8.6	65.0 ± 8.4	0.297	−0.046	64.5 ± 8.5	64.7 ± 8.3	0.709	−0.022
Male gender, *n* (%)	481 (63.1)	850 (57.7)	0.013	0.112	403 (63.0)	403 (63.0)	1.000	−0.001
BMI, mean ± SD kg/m^2^	23.3 ± 3.8	23.1 ± 3.8	0.208	0.057	23.1 ± 3.6	23.4 ± 3.8	0.161	0.079
NYHA class (≧Ⅲ), *n* (%)	267 (35.0)	643 (43.6)	<0.001	−0.176	231 (36.0)	228 (35.6)	0.861	0.008
STS SCORE, (IQR)	1.57 (1.02–2.81)	1.82 (1.06–3.33)	0.005	−0.072	1.56 (1.03–2.77)	1.77 (0.99–3.09)	0.261	−0.003
Euro SCORE, (IQR)	1.76 (1.08–3.40)	2.02 (1.2–3.87)	<0.001	−0.077	1.73 (1.07–3.40)	1.89 (1.1–3.5)	0.169	−0.002
LVEF, mean ± SD %	51.6 ± 15.4	50.5 ± 16.1	0.096	0.075	51.5 ± 15.3	51.5 ± 15.8	0.959	−0.003
Comorbidity, *n* (%)								
Hyperlipidemia	746 (97.9)	1,439 (97.6)	0.680	0.018	631 (98.6)	628 (98.1)	0.509	0.037
Hypertension	746 (97.9)	1,455 (98.7)	0.143	−0.063	631 (98.6)	630 (98.4)	0.817	0.013
Diabetes mellitus	358 (47.0)	709 (48.1)	0.616	−0.022	315 (49.2)	305 (47.7)	0.576	0.031
Chronic renal disease (Cr ≧ 1.5)	119 (15.6)	315 (21.4)	0.001	−0.151	112 (17.5)	96 (15.0)	0.225	0.064
Dialysis	99 (13.0)	263 (17.8)	0.003	−0.132	89 (13.9)	74 (11.6)	0.205	0.071
COPD	78 (10.2)	151 (10.2)	0.995	0.000	67 (10.5)	67 (10.5)	1.000	0.001
Cerebral vascular accident	57 (7.48)	76 (5.16)	0.028	0.096	46 (7.19)	45 (7.03)	0.913	0.007
Peripheral arterial disease	117 (15.4)	200 (13.6)	0.251	0.048	92 (14.4)	88 (13.8)	0.748	0.014
STEMI	142 (18.6)	231 (15.7)	0.075	0.080	108 (16.9)	105 (16.4)	0.822	0.013
Recent myocardial infarction	431 (56.6)	864 (58.6)	0.351	−0.042	359 (56.1)	344 (53.8)	0.399	0.047
One vessel disease	64 (8.40)	15 (1.02)	<0.001	0.354	18 (2.81)	14 (2.19)	0.474	0.040
Double vessel disease	189 (24.8)	143 (9.7)	<0.001	0.406	118 (18.44)	119 (18.6)	0.943	−0.007
Triple vessel disease	510 (66.9)	1,307 (88.7)	<0.001	−0.540	503 (78.6)	507 (79.2)	0.784	−0.012
Left main trunk lesions	320 (42.0)	540 (36.6)	0.014	0.110	267 (41.7)	267 (41.7)	1.000	0.000
Preoperation PCI	77 (10.1)	98 (6.7)	0.004	0.126	59 (9.2)	56 (8.75)	0.769	0.017
Preoperation IABP	96 (12.6)	288 (19.5)	<0.001	−0.191	87 (13.6)	93 (14.5)	0.630	−0.031
Urgency, *n* (%)								
Elective	569 (74.7)	1,128 (76.6)	0.331	−0.041	488 (76.3)	490 (76.6)	0.895	−0.005
Urgent	187 (24.5)	324 (22.0)	0.172	0.059	147 (22.9)	148 (23.1)	0.947	−0.007
Emergent	4 (0.52)	9 (0.61)	1.000	−0.011	3 (0.47)	1 (0.16)	0.624	0.056
Salvage	6 (0.79)	20 (1.36)	0.234	−0.055	5 (0.78)	3 (0.47)	0.726	0.040

BMI, body mass index; NYHA, New York Heart Association; STS, Society of Thoracic Surgeons; Euro SCORE, European System for Cardiac Operative Risk Evaluation; LVEF, left ventricular ejection fraction; COPD, chronic obstructive pulmonary disease; STEMI, ST-elevation myocardial infarction; PCI, percutaneous coronary intervention; IABP, intra-aortic balloon pumping.

**Table 2:** Derivation of propensity score equation from pretreatment covariates under multivariable binary logistic regression.

Pretreatment covariates	Coefficient	95% confidence interval	*p*-value
Age, year	−0.05	−0.02, 0.07	0.411
Male gender	0.28	0.08, 0.48	0.005
BMI, kg/m^2^	0.11	−0.01, 0.04	0.378
NYHA class (≧Ⅲ), *n* (%)	−0.37	−0.60, −0.14	0.002
Comorbidity, *n* (%)			
Hyperlipidemia	0.28	−0.44, 1.00	0.447
Hypertension	−0.57	−1.34, 0.20	0.145
Diabetes mellitus	0.19	−0.01, 0.39	0.050
Chronic renal disease (Cr ≧ 1.5)	−0.26	−0.62, 0.10	0.153
Dialysis	−0.12	−0.52, 0.28	0.548
COPD	0.07	−0.26, 0.39	0.687
Cerebral vascular accident	0.45	0.06, 0.84	0.024
Peripheral arterial disease	0.16	−0.13, 0.45	0.277
STEMI	0.08	−0.18, 0.34	0.553
Recent myocardial infarction	−0.15	−0.36, 0.06	0.163
One vessel disease	3.20	1.60, 4.80	<0.001
Double vessel disease	2.03	0.50, 3.56	0.009
Triple vessel disease	0.81	−0.71, 2.33	0.296
Left main trunk lesions	0.29	0.10, 0.48	0.004
Preoperation PCI	0.32	−0.03, 0.67	0.073
Preoperation IABP	−0.38	−0.68, −0.09	0.011
Echocardiography			
LVEF, %	−0.10	−0.75, 0.55	0.76
Urgency, *n* (%)			
Elective	0.15	−0.92, 1.23	0.779
Urgent	0.51	−0.56, 1.57	0.349
Emergent	0.11	−1.56, 1.77	0.898
Salvage	0.05	−1.38, 1.47	0.949

BMI, body mass index; NYHA, New York Heart Association; COPD, chronic obstructive pulmonary disease; STEMI, ST-elevation myocardial infarction; PCI, percutaneous coronary intervention; IABP, intra-aortic balloon pumping; LVEF, left ventricular ejection fraction.

## 3 Results

Preoperative patient characteristics before and after matching are presented in Table 1. In the order of NT group and A group, there were 481 (63.1%) and 850 (57.7%) males, with mean ages of 64.4 ± 8.6 and 65.0 ± 8.4 years in the unmatched cohort, and 403 (63.0%) and 403 (63.0%) males, with mean ages of 64.5 ± 8.5 and 64.7 ± 8.3 years in the matched cohort. After matching, all categories showed a standardized mean difference below 0.1.

Intraoperative results between the two groups are presented in Table 3. After matching, the use of intraoperative blood transfusion was significantly higher in the A group (65.8% vs. 77.7%, *p* < 0.001).

**Table 3:** Operative data.

	Entire cohort			Matched cohort		
	NT group (*n* = 762)	A group (*n* = 1,474)	*p*-value	NT group (*n* = 640)	A group (*n* = 640)	*p*-value
Early-term operation in this study, *n* (%)	663 (87.0)	919 (62.4)	<0.001	555 (86.7)	428 (66.9)	<0.001
Operating time, mean ± SD min	236.3 ± 71.4	241.1 ± 61.4	0.097	246.2 ± 68.9	239.4 ± 62.9	0.9662
Total grafts number, mean ± SD	2.5 ± 1.0	3.0 ± 0.8	<0.001	2.7 ± 0.9	2.9 ± 0.8	<0.001
Number of distal anastomoses, mean ± SD	2.8 ± 1.1	3.4 ± 0.9	<0.001	3.0 ± 1.1	3.3 ± 0.9	<0.001
Over two atrial grafts, *n* (%)	539 (70.7)	607 (41.2)	<0.001	495 (77.3)	280 (43.8)	<0.001
Total arterial revascularization, *n* (%)	531 (69.7)	66 (4.5)	<0.001	433 (67.7)	39 (6.1)	<0.001
Endarterectomy, *n* (%)	10 (1.3)	46 (3.1)	0.009	9 (1.4)	15 (2.3)	0.303
Transfusion, *n* (%)	485 (63.7)	1,205 (81.8)	<0.001	421 (65.8)	497 (77.7)	<0.001
Complete revascularization, *n* (%)	552 (72.4)	1,163 (76.9)	0.001	476 (74.4)	492 (76.9)	0.298
Conversion to CPB, *n* (%)	4 (0.5)	25 (1.7)	0.020	4 (0.6)	11 (1.7)	0.116
Left mini-thoracotomy, *n* (%)	320 (42.0)	540 (36.6)	0.014	144 (22.5)	45 (7.03)	<0.001
Graft, *n* (%)						
LITA	750 (98.4)	1,402 (95.1)	<0.001	632 (98.8)	611 (95.5)	<0.001
RITA	458 (60.1)	160 (10.9)	<0.001	425 (66.4)	79 (12.3)	<0.001
Radial artery	245 (32.2)	493 (33.5)	0.537	226 (35.3)	221 (34.5)	0.769
Gastroepiploic artery	177 (23.2)	11 (0.8)	<0.001	172 (26.8)	4 (0.6)	<0.001
Saphenous vein	210 (27.6)	1,403 (95.2)	<0.001	192 (30.0)	601 (93.9)	<0.001

CPB, cardiopulmonary bypass; LITA, left internal thoracic artery; RITA, right internal thoracic artery.

The period from April 2011 to March 2017 was defined as the early-term operation, which was significantly higher in the NT group (86.7% vs. 66.9%, *p* < 0.001). The number of distal anastomoses (3.0 ± 1.1 vs. 3.3 ± 0.9, *p* < 0.001) and graft number (2.7 ± 0.9 vs. 2.9 ± 0.8, *p* < 0.001) were significantly higher in the A group compared to the NT group. Multiple arterial grafting rate was higher in the NT group (NT vs. A; more than two arterial grafts, 77.3% vs. 43.8%, *p* < 0.001; total arterial revascularization, 67.7% vs. 6.1%, *p* < 0.001). The use of the left internal thoracic artery (98.8% vs. 95.5%, *p* < 0.001), right internal thoracic artery (66.4% vs. 12.3%, *p* < 0.001), and gastroepiploic artery (26.8% vs. 0.6%, *p* < 0.001) were respectively significantly higher in the NT group, whereas the use of the saphenous vein (30.0% vs. 93.9%, *p* < 0.001) was higher in the A group.

Table 4 shows the postoperative results between NT and A groups. There was no difference in new stroke rates: 1.0% vs. 1.2% (*p* = 0.624) in the before-matched cohort and 0.9% vs. 1.3% (*p* = 0.789) in the matched cohort in the NT and A groups, respectively. There was also no difference in 30-day mortality; 1.7% vs. 2.9% (*p* = 0.069) in the before-matched cohort and 1.7% vs. 2.0% (*p* = 0.837) in the matched cohort in NT and A groups, respectively. After PSM, the rates of new-onset AF (18.6% vs. 24.4%, *p* = 0.012) and median drain contents (IQR) [400 (300–500) ml vs. 400 (320–550) ml, *p* = 0.021] were significantly lower in the NT group compared to the A group. There were no statistically significant differences in the duration of intensive care unit (ICU) or hospital stay, other major complications, or MACCE event rates between the two groups.

**Table 4:** Postoperative short outcomes.

	Entire cohort			Matched cohort		
	NT group (*n* = 762)	A group (*n* = 1,474)	*p*-value	NT group (*n* = 640)	A group (*n* = 640)	*p*-value
Median ICU stay (IQR), days	2 (1–2)	2 (1–2)	0.672	2 (1–2)	2 (1–2)	0.377
Median hospital stay (IQR), days	5 (5–6]	5 (5–7)	0.144	5 (5–6)	5 (5–6)	0.648
Early extubation (≦24 h), *n* (%)	674 (88.5)	1,291 (87.6)	0.552	562 (87.8)	573 (89.5)	0.332
Median drain contents, ml (IQR)	400 (300–500)	400 (320–550)	<0.001	400 (300–500)	400 (320–550)	0.021
30 days mortality, *n* (%)	13 (1.7)	44 (2.9)	0.069	11 (1.7)	13 (2.0)	0.837
Early-term postoperative complications, *n* (%)						
Postoperative new stroke	8 (1.0)	19 (1.2)	0.624	6 (0.9)	8 (1.3)	0.789
New dialysis	1 (0.1)	21 (1.4)	0.003	1 (0.2)	6 (0.9)	0.124
New-onset atrial fibrillation/flutter	150 (19.7)	397 (26.9)	<0.001	119 (18.6)	156 (24.4)	0.012
Infection of wound	7 (0.9)	12 (0.8)	0.799	5 (0.8)	4 (0.6)	1.000
Reoperation of bleeding	8 (1.1)	31 (2.1)	0.071	5 (0.8)	17 (2.8)	0.235
MACCE long-term, *n* (%)	70 (9.2)	205 (13.9)	0.001	59 (9.2)	70 (10.9)	0.307
Cardiac death	45 (5.9)	140 (9.5)	0.003	40 (6.3)	45 (7.0)	0.654
Long-term stroke event	17 (2.2)	44 (3.0)	0.299	12 (1.9)	18 (2.8)	0.356
Heart failure requiring hospitalization	14 (1.8)	36 (2.4)	0.359	11 (1.7)	4 (0.6)	0.116
Postoperative myocardial infarction	8 (1.1)	31 (2.1)	0.071	6 (0.9)	14 (2.2)	0.112
Repeat revascularization	3 (0.47)	9 (1.41)	0.169	3 (0.47)	9 (1.41)	0.144

ICU, intensive care unit; MACCE, major adverse cardiac or cerebrovascular events.

**Figure 1:**
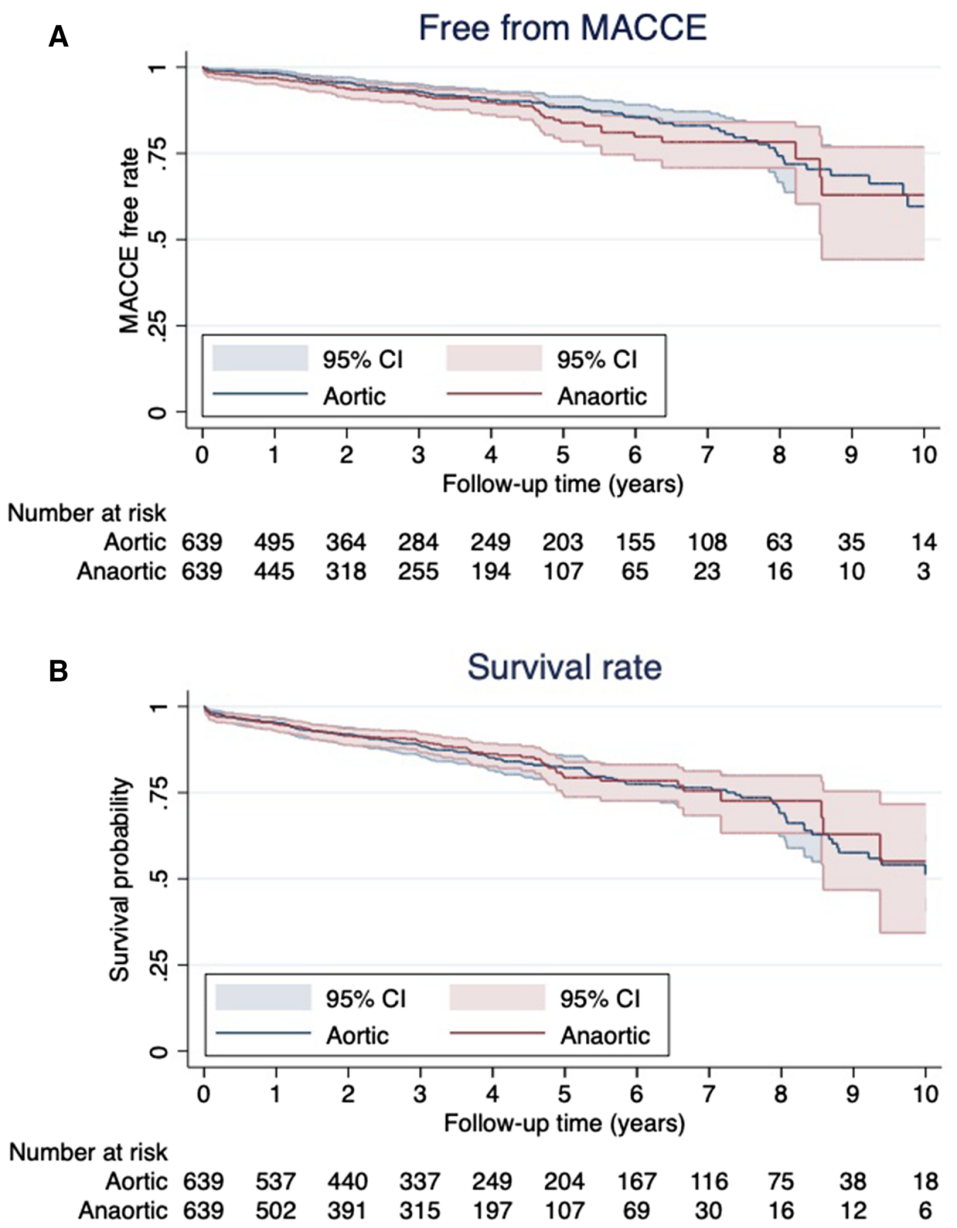
Kaplan–Meier analysis for major adverse cardiac or cerebrovascular event (MACCE)-free rate (**A**) and survival rate (**B**).

**Figure 2:**
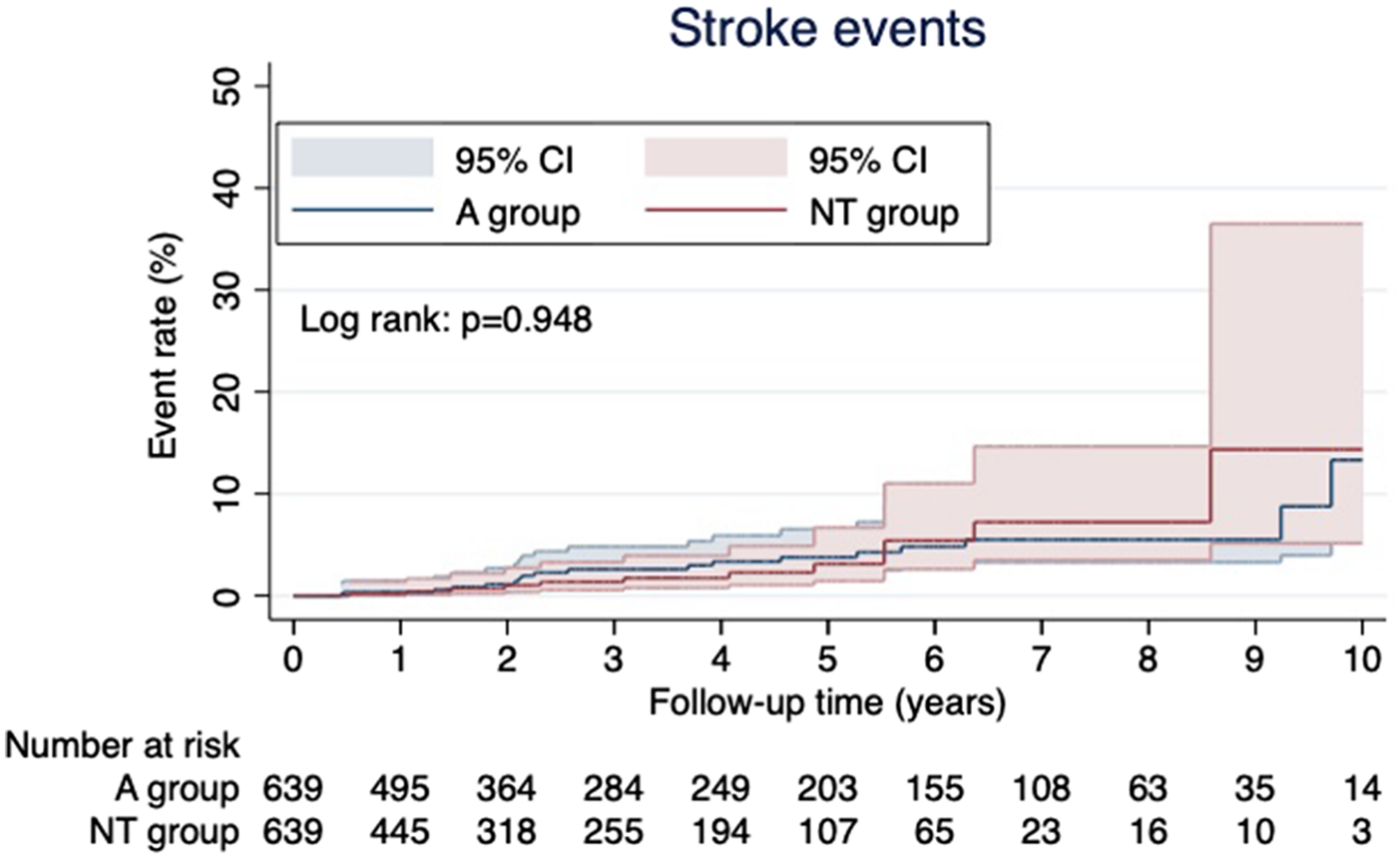
Kaplan–Meier analysis for stroke events during the post-OPCAB follow-up period.

After PSM, the Kaplan–Meier curves of the postoperative MACCE-free rate and survival rate are shown in Figure 1. There were no significant differences in each item between the two groups (MACCE-free rate, *p* = 0.228; survival rate, *p* = 0.783). Additionally, the anaortic technique did not show a difference in long-term stroke events after OPCAB (*p* = 0.948, Figure 2).

The univariable and multivariable risk regression analyses performed to identify risk factors for new stroke are presented in Table 5. The univariable analysis indicated that the risk factors for new strokes included a history of cerebral vascular accident (CVA), peripheral vessel disease (PVD), salvage operative status, reoperation due to postoperative bleeding, and postoperative new-onset AF. However, the anaortic technique was not identified as an independent factor negatively associated with new stroke events (OR = 0.81, 95% CI = 0.35–1.86, *p* = 0.624). The history of PVD (OR = 2.38, 95% CI = 1.02–5.56, *p* = 0.045) and new-onset AF (OR = 4.45, 95% CI = 1.98–10.01, *p* < 0.001) were significant risk factors in the multivariable analysis of these factors.

**Table 5:** Univariable and multivariable analyses for factors associated with new stroke.

Variable	Univariable analysis			Multivariable analysis		
	OR	95% CI	*p*-value	OR	95% CI	*p*-value
Early-term operation in this study	0.54	0.18–1.62	0.272			
Preoperative factor						
Age	1.03	0.99–1.08	0.149			
Male gender	1.16	0.53–2.54	0.715			
Body mass index	0.99	0.90–1.10	0.863			
NYHA class (≧Ⅲ)	1.17	0.54–2.51	0.690			
Diabetes mellitus	1.88	0.855–4.12	0.116			
Chronic renal disease (C r ≧ 1.5)	1.46	0.61–3.48	0.392			
Dialysis	1.49	0.60–3.71	0.396			
COPD	2.01	0.76–5.37	0.162			
Cerebral vascular accident	3.70	1.38–9.92	0.009	2.44	0.83–7.11	0.103
Peripheral arterial disease	3.64	1.65–8.03	<0.001	2.42	1.04–5.66	0.041
STEMI	0.40	0.93–1.68	0.209			
Recent myocardial infarction	2.09	0.88–4.97	0.881			
Left main trunk lesions	1.49	0.70–3.20	0.301			
Double vessel disease	1.65	0.66–4.12	0.283			
Triple vessel disease	0.80	0.32–2.01	0.641			
Preoperative PCI	0.94	0.22–4.01	0.935			
Preoperative IABP	1.38	0.55–3.45	0.487			
LVEF	0.18	0.02–2.01	0.165			
Elective	0.54	0.24–1.18	0.120			
Urgent	1.18	0.50–2.82	0.702			
Emergent	7.04	0.88–56.16	0.065	5.30	0.62–45.27	0.127
Salvage	7.28	1.63–32.49	0.009	2.65	0.53–13.14	0.233
Intraoperative and postoperative factors						
Operation time	1.00	0.99–1.01	0.722			
Aorta no-touch technique	0.81	0.35–1.86	0.624			
Left mini-thoracotomy	0.52	0.12–2.22	0.380			
Pump conversion	3.00	0.39–22.85	0.290			
Reoperation of bleeding	4.70	1.07–20.56	0.040	3.18	0.69–14.74	0.138
New-onset atrial fibrillation/flutter	5.39	2.45–11.83	<0.001	4.29	1.90–9.69	<0.001

NYHA, New York Heart Association; COPD, chronic obstructive pulmonary disease; STEMI, ST-elevation myocardial infarction; PCI, percutaneous coronary intervention; IABP, intra-aortic balloon pumping; LVEF, left ventricular ejection fraction.

## 4 Discussion

Contrary to previous reports (1–3), there was no association observed between postoperative stroke and aortic manipulation in OPCAB surgery in our single-center study. Moreover, anaortic OPCAB had no positive impact on long-term stroke prevention.

### 4.1 Do anaortic techniques reduce new stroke events?

Recent reports have claimed that there is little difference in outcome for most patients between OPCAB and conventional CABG (5, 6). However, it is also well accepted that OPCAB is valuable in reducing the risk of stroke and preserving the heart and renal function in higher-risk patients and that the most reliable tactics for avoiding perioperative stroke are the maintenance of blood pressure and the avoiding aortic manipulation (7–9). For these reasons, the anaortic technique OPCAB has been hailed for preventing the incidence of new stroke events. Pawliszak et al. (3) reported a meta-analysis comparing anaortic technique and proximal anastomosis devices with side-clamp OPCAB3 involving 25,163 patients across 18 studies. The anaortic technique demonstrated a decrease in postoperative cerebrovascular events of nearly 60%, compared to side-clamp OPCAB, and this benefit was consistent across different patient risk levels. In another study, Zhao et al. (9) conducted a meta-analysis comparing post-CABG outcomes, incorporating data from 13 studies with 37,720 patients. They concluded that anaortic OPCAB was the most effective treatment for decreasing the postoperative risk of stroke. However, it was not evident in our study that anaortic OPCAB reduces the incidence of perioperative strokes (NT 0.9% vs. A 1.3%, *p* = 0.789). In addition, the univariable analysis indicated that the risk factors for new strokes were not associated with the aortic technique. Generally, the important and major risk factors for stroke after CABG were as follows: old age, aortic atheromatous disease, aortic manipulation, diabetes, female sex, hypertension, PVD, previous neurological injury, symptomatic carotid stenosis, and use of cardiopulmonary bypass (CPB) (10, 11). Our study demonstrated that a history of PVD (OR = 2.38, 95% CI = 1.02–5.56, *p* = 0.045) and new-onset AF (OR = 4.45, 95% CI = 1.98–10.01, *p* < 0.001) were significant risk factors in the multivariable analysis. This implies that adopting the anaortic technique may not be universally consequential in averting the onset of new strokes. While the anaortic technique need not be performed routinely for the sake of reducing perioperative stroke risk, it should always remain a viable option when performing OPCAB for patients with a high risk of stroke.

### 4.2 Mid- and long-term benefits of anaortic OPCAB technique

When anaortic techniques are performed, the internal thoracic arteries are commonly used, and reap the benefits of multiple artery grafts or total arterial reconstruction (TAR) (12–14). However, it has not been reported that the anaortic technique improves the long-term survival rate or the incidence of MACCE. Furukawa et al. (15) reported a prospective study comparing mid-term outcomes of anaortic OPCAB (*n* = 1,233), clampless OPCAB (*n* = 2,310), and conventional CABG (*n* = 1,879). Their study showed no difference in MACCE nor mortality in mid-term outcomes between the three groups. In our study, despite multiple arterial grafting rates being higher in the NT group than in the A group (aortic manipulation group), there were no significant differences in either the MACCE-free (*p* = 0.228) rates or survival rates (*p* = 0.783) between the two groups. In addition, the anaortic technique did not reduce long-term stroke events after OPCAB (*p* = 0.948). Albert et al. (16) elucidated the impact of the anaortic technique on post-CABG strokes (*n* = 4,485) compared with on-pump CABG (*n* = 8,794). They reported anaortic OPCAB resulted in a significant reduction of the overall postoperative risk of stroke (0.45% vs. 1.28% in CPB patients; *p* = 0.0001), but delayed strokes were not significantly reduced [0.37% (95% CI, 0.18%–0.57%) vs. 0.46% (95% CI, 0.24%–0.67%)] in anaortic OPCAB vs. CPB patients, respectively (*p* = 0.5749). These results show that although anaortic OPCAB is not beneficiary in preventing long-term strokes, it reduces the risk of strokes caused by acute perioperative embolism from the thoracic aorta. However, anaortic techniques may supply long-term benefits to patients in other aspects.

### 4.3 Road to performing anaortic OPCAB

According to recent guidelines (17, 18), multiple arterial revascularizations, application of different appropriate modalities, and unclamping the ascending aorta to avoid stroke are all recommended strategies. The whole operation requires various procedures: (1) a skeletonized technique for harvesting the arterial conduits, (2) a traction technique to create the best exposure without hemodynamic compromise using appropriate instruments, (3) generation of composite grafts followed by optimal alignment of the conduits without kinking, and (4) a technique with a high level of anastomoses. All of these procedures must be performed with the heart still beating, which is not the case in conventional CABG. Due to the high level of technicality required, it is challenging for a surgeon who is not familiar with the knacks and pitfalls of anaortic OPCAB to perform and complete the procedure in its entirety safely. To perform anaortic technique reproducibly and with satisfactory outcomes, it is important to incorporate OPCAB into regular practice, to enable not only the surgeons but also the medical staff, to acquire abundant experience of OPCAB procedures (19). In our institution, the anaortic technique has been established as a method of choice for all OPCAB patients, so operation times and major complication rates were not significantly different.

### 4.4 Study limitations

To point out some important limitations, first, this is a retrospective, non-randomized analysis from a single medical center. Second, PSM was based on preoperational patient characteristics, with several unmeasured confounders. In addition, although we included many patients in this study, it remains underpowered to properly assess the true effect of anaortic OPCAB on the studied outcome because the new stroke rate was a rare complication (approximately 1%). Third, there was a selection bias in favor of choosing the anaortic technique, which tended to be more frequently employed in patients with aortic disease. In this study, procedures in the A group utilized anastomosis assist devices while all anastomoses were done purely manually in the NT group. Additionally, the application of anastomosis assist devices was constrained, primarily due to a lack of insurance reimbursement in Thailand. Fourth, we did not conduct consistent preoperative assessments, such as head lack of magnetic resonance imaging (MRI) or CT scans, for brain imaging in many patients. Consequently, the information regarding previous CVA may not be accurate. Finally, the asymptomatic strokes were not included in our analysis due to the lack of routine postoperative head CT scans.

## 5 Conclusion

The present study did not find the anaortic technique to reduce the perioperative risk of stroke in OPCAB. Hence, further large studies are needed to identify patient cohorts in which anaortic OPCAB is significantly beneficial.

## Data Availability

The raw data supporting the conclusions of this article will be made available by the authors, without undue reservation.
